# Likeability of Garden Birds: Importance of Species Knowledge & Richness in Connecting People to Nature

**DOI:** 10.1371/journal.pone.0141505

**Published:** 2015-11-11

**Authors:** Daniel T. C. Cox, Kevin J. Gaston

**Affiliations:** Environment & Sustainability Institute, University of Exeter, Penryn, Cornwall, United Kingdom; Cornell University, UNITED STATES

## Abstract

Interacting with nature is widely recognised as providing many health and well-being benefits. As people live increasingly urbanised lifestyles, the provision of food for garden birds may create a vital link for connecting people to nature and enabling them to access these benefits. However, it is not clear which factors determine the pleasure that people receive from watching birds at their feeders. These may be dependent on the species that are present, the abundance of individuals and the species richness of birds around the feeders. We quantitatively surveyed urban households from towns in southern England to determine the factors that influence the likeability of 14 common garden bird species, and to assess whether people prefer to see a greater abundance of individuals or increased species richness at their feeders. There was substantial variation in likeability across species, with songbirds being preferred over non-songbirds. Species likeability increased for people who fed birds regularly and who could name the species. We found a strong correlation between the number of species that a person could correctly identify and how connected to nature they felt when they watched garden birds. Species richness was preferred over a greater number of individuals of the same species. Although we do not show causation this study suggests that it is possible to increase the well-being benefits that people gain from watching birds at their feeders. This could be done first through a human to bird approach by encouraging regular interactions between people and their garden birds, such as through learning the species names and providing food. Second, it could be achieved through a bird to human approach by increasing garden songbird diversity because the pleasure that a person receives from watching an individual bird at a feeder is dependent not only on its species but also on the diversity of birds at the feeder.

## Introduction

Interacting with nature is now widely recognised as providing many health and well-being benefits to people living in urban environments, particularly in westernised societies [1]. These can include positive influences on physical health (e.g. [2,3]), on psychological health (e.g. [1–8]) and on social health (e.g. [8,9]). The majority of studies have considered benefits in terms of interactions with the natural environment at large, typically in the form of green spaces, in what has been termed ‘nature doses’ [10,11]. However, there is limited understanding of the individual components of nature that provide these benefits [6,12]. Nonetheless, there is evidence that watching birds and listening to bird song can have positive psychological effects [13,14]. Understanding how the abundance and richness of important well-being benefit providers, such as birds, contributes towards the overall benefits from nature may be key in learning how best to utilise these benefits.

For many people living in urban areas their interactions with wild birds may form the main, or at least the most readily recognised, wildlife interactions that they experience in daily life [15]. As a consequence, the provision of food for birds in domestic gardens has the potential to be an important vector for delivery of well-being benefits [16,17]. Garden bird feeding is the most common form of wildlife gardening, and one that continues to grow in popularity, with about a half of urban households in Western countries for which there are data putting out food on a regular basis (estimated from [17–22]). A bird feeder attracts birds to domestic gardens, attracting a greater number and diversity of species [23,24]. Thus bird feeders can be seen to alter the flow of associated well-being benefits into gardens. They also provide focal points for the delivery of these benefits because they are where people expect to see birds and so are more likely to watch them for longer periods and at closer proximity.

Evidence suggests that watching birds at garden feeders gives people an increased sense of psychological well-being because it provides them with a feeling of pleasure [17,25] and of being connected to nature [16]. This is important because how a person relates to nature has been shown to be a good predictor of general life happiness [26]. However, not all species are viewed equally, with some being preferred over others [27,28]. A species ‘likeability’ can influence factors ranging from the allocation of funds for its conservation [29] and the level of concern over its welfare [30], to how much time people spend watching its behaviour [31] or the money they spend on supplying it with food and related products (e.g. [17]). How appealing a person finds a species, and how satisfied they feel when they see a particular species or group of species is likely to determine the levels of pleasure that they feel from watching it [13].

The varying degrees of pleasure people feel from seeing birds at their feeders may correspond to both the number of individuals (i.e. abundance) and species (i.e. richness) of birds present (termed the ‘intensity quantity’ and the ‘intensity quality’, respectively, of the nature dose [10,11]). Indeed, within urban green space, species richness, or perceived species richness, has been found to be positively correlated with increased psychological well-being [4,18,32]. Bird feeders can be seen to provide an important tool for highlighting species diversity, because they attract birds to a known location where multiple species are often easily visible. So although perceptions of species richness within the wider environment can be highly inaccurate [6,33,34], bird feeders provide a focal location where people are often likely to be able to distinguish between different species and so appreciate diversity even if they do not know the formal names of the species. Teasing apart the role that varying numbers of individuals and species richness play in providing well-being benefits will help stakeholders in urban planning and land management, and conservation initiatives to maximise the connection to nature that people feel when they interact with birds. Scaled-up this has the potential to increase the benefits provided by birds in and around domestic gardens. For example, if people prefer to see increased species richness at their feeders then conservation organisations should encourage provision of a greater and consistent range of foods at private feeding stations.

We used a quantitative survey exploring householders’ knowledge of, and attitudes towards, garden birds and bird feeding to explore two key questions: 1) What factors influence the likeability of 14 common garden bird species?, and 2) Do people prefer species richness (nature quality) or individual abundance (nature quantity) at their bird feeders?

## Materials and Methods

### Experimental design

We surveyed garden bird feeding activities and perceptions of common garden bird species using a questionnaire approach across three English towns located, in close proximity (~60 km to the north of London, UK): Milton Keynes (52°02’N, 0°45’W), Luton (51°53’N, 0°25’W) and Bedford (N52°58’N, 0°28’W). These each have sizeable human populations of, respectively, c. 230,00, c. 240,000, and c. 160,000 (2011 Census, UK). Two general survey methods were used. First, between November 2013 and February 2014, 20 households were selected at random in each of the three towns. A researcher knocked on the doors of the houses and asked one member of the household to complete the questionnaire. The survey participant in each household was also asked to enlist two other known households from within ~500m to participate in the survey. Potential participants were contacted by email or phone and the questionnaire was delivered by hand. Second, between March and July 2014 up to ten streets in each town were selected at random. A researcher then knocked on the doors of all houses with evidence that someone was home, e.g. from a car in the drive. The project was explained to the resident, who was then asked to complete a questionnaire in his or her own time. In order to minimize possible bias resulting from certain groups being more likely to be at home, different streets were targeted at different times of day either late morning (11:00 to 13:00), mid afternoon (14:30 to 16:00) or late afternoon (17:00 to 18:30). Surveys were conducted at both weekdays and at weekends. For both survey methods a first attempt to collect the questionnaire was made two days after delivery, and if unsuccessful a subsequent attempt was made two days after that. One-hundred and forty responses were collected by the first survey method, and 191 by the second.

### Ethics clearance

This research was conducted in accordance with the University of Exeter Biosciences ethical review committee, project number 2013/320. Before completing the survey respondents were asked to provide written consent by checking a box stating their agreement to participate in the survey. Respondents were also asked to confirm that they were over 18 years of age. On the written consent form, participants were told that data would remain anonymous and would be protected and stored in a secured format. There is a electronic log of consent procedure to document the process. This procedure was also approved by the Biosciences ethical review committee, University of Exeter.

### Questionnaire design

We developed a questionnaire to explore people’s knowledge and experience of, and attitudes towards, garden birds and bird feeding. The questionnaire took approximately six minutes to complete and consisted of close-ended questions. Only those questions used in the analyses reported here are discussed.

To explore varying perceptions across species we asked people to rate on a scale of 1–5, from strongly dislike to strongly like, how appealing they found each of 14 pictures of common UK garden bird species, and to add the name of the species if they knew it (Table A in S1 File). If a score was given but no name it was assumed that this was not known. If a partial name was given, but the species could be identified from the name then the respondent was scored as knowing the species name (e.g. ‘sparrow’ instead of ‘house sparrow’). We then developed two, two-level factors of whether the species was a songbird or non-songbird, and if the person could name the species (yes or no). We calculated a continuous variable of the number of species that each respondent could name.

To explore whether people preferred to see species richness (nature intensity quality) or individual abundance (nature intensity quantity) of birds at their feeders, respondents were shown six pictures of varying numbers of individuals and songbird species at a bird feeder (two, five or eight individuals or species; Figure A in S1 File). Different garden bird species vary in colour and size and so respondents are likely to be able to tell them apart when they see them at a feeder and so detect diversity, even with limited knowledge of the species. As such, to avoid possible biases resulting from people’s preferences for some bird species, different numbers and colours of a single drawing of a small bird were used, where different colours represented different bird species. Respondents were then asked to rank in order from 1–6 how satisfied they were with each picture, with 6 being the picture that they were most satisfied with and 1 that they were least satisfied with.

We also collected data on the sociodemographic status of each respondent, how regularly they provided food to birds (if at all), the approximate periods of the day they noticed birds and how connected they felt to nature when they saw birds in their garden (see Table B in S1 File). Prior to statistical analysis we created a three-level factor pertaining to how regularly food was provided for birds: regularly (those that replied daily or weekly), irregularly (those that replied monthly or less than once a month) or never (those that do not feed birds). As a measure of people’s awareness of the birds around where they live and work, respondents were asked to select one or more periods during the day when they usually noticed birds (day was divided into four approximately equal periods; morning, lunchtime, afternoon and evening). We constructed a fourth factor on a scale of 0–4 according to the periods of their average day people reported noticing birds. Those that ticked ‘I don’t notice birds’ were given a score of zero. As an additional part of the survey we asked people to rate on a scale of one to five from strongly disagree to strongly agree, to what extent they agreed with the statement ‘I feel connected to nature when I see birds in my garden’. This was then included as an ordered factor. We created a sixth factor of respondent’s gender (male/female). Respondents were asked their age within a five-year window, and finally we then developed a seventh factor with ages pooled from 20 to 40 years, 40 to 60 years and >60 years. These five factors are summarised in Table C in S1 File.

### Statistical analysis

All analysis was conducted in R 3.1.2 [35]. We pooled responses from both methods of data collection (Appendix A in S1 File) and from the three towns. For any completed questionnaire, if any of the questions were incomplete, then that respondent’s question was removed from the analysis. Generalized Variance Inflation Factors (GVIFs) were used to check for multi-collinearity between factors, and found to be within acceptable norms, with all GVIFs <1.1. We created a categorical response variable by pooling likeability scores across species and then including the species name as a random effect. To determine whether levels of bird feeding, bird awareness, a person’s connection to nature when they see birds, gender, age, whether the bird was a songbird or not and if a person could name the species were important predictors of a species likeability we built mixed-effect ordinal regression models using the ‘ordinal’ package [36]. We then applied an Information Theoretic approach that simultaneously evaluates hypotheses by balancing between model complexity and goodness of fit [37]. We used the ‘MuMIn’ package [38] to produce all subsets of models based on the global model and rank them based on AICc. Following [39], and to be 95% sure that the most parsimonious models were maintained within the best supported model set, we retained all models where **Δ**AIC_c_ < 6. We then used model-averaging to produce the average parameter estimates and relative importance (RI) of each parameter [37].

We carried out a linear regression on the number of species that a person could correctly identify against their self-reported connection to nature when they watched birds in their garden, how regularly they fed birds and for what proportion of the day they noticed birds. We also incorporated age and gender as factors in the model.

To explore whether people preferred to see species richness (nature quality) or individual abundance (nature quantity) at their bird feeders we ranked the pictures of varying numbers of songbird individuals and species from 1–6 relative to their mean scores, before plotting the mean and variance of each picture in their rank order.

## Results

### Respondents

A total of 331 questionnaires were completed and used in the analysis. For the first survey method we received a response rate of 94%. For the second survey method, 90% agreed to participate in the survey, of these 87% completed the survey giving an overall return rate of 78%. There was an over representation of female respondents (56% compared to 51% in Buckinghamshire and Bedfordshire county’s, 2011 Census) and of respondents over 60 years (42% compared to 28% in Buckinghamshire and Bedfordshire county’s, 2011 Census; Table B in S1 File). We found that 83% of households put out bird food, with 72% of those feeding birds doing so regularly (Table C in S1 File).

### Likeability of garden birds

**Fig 1:**
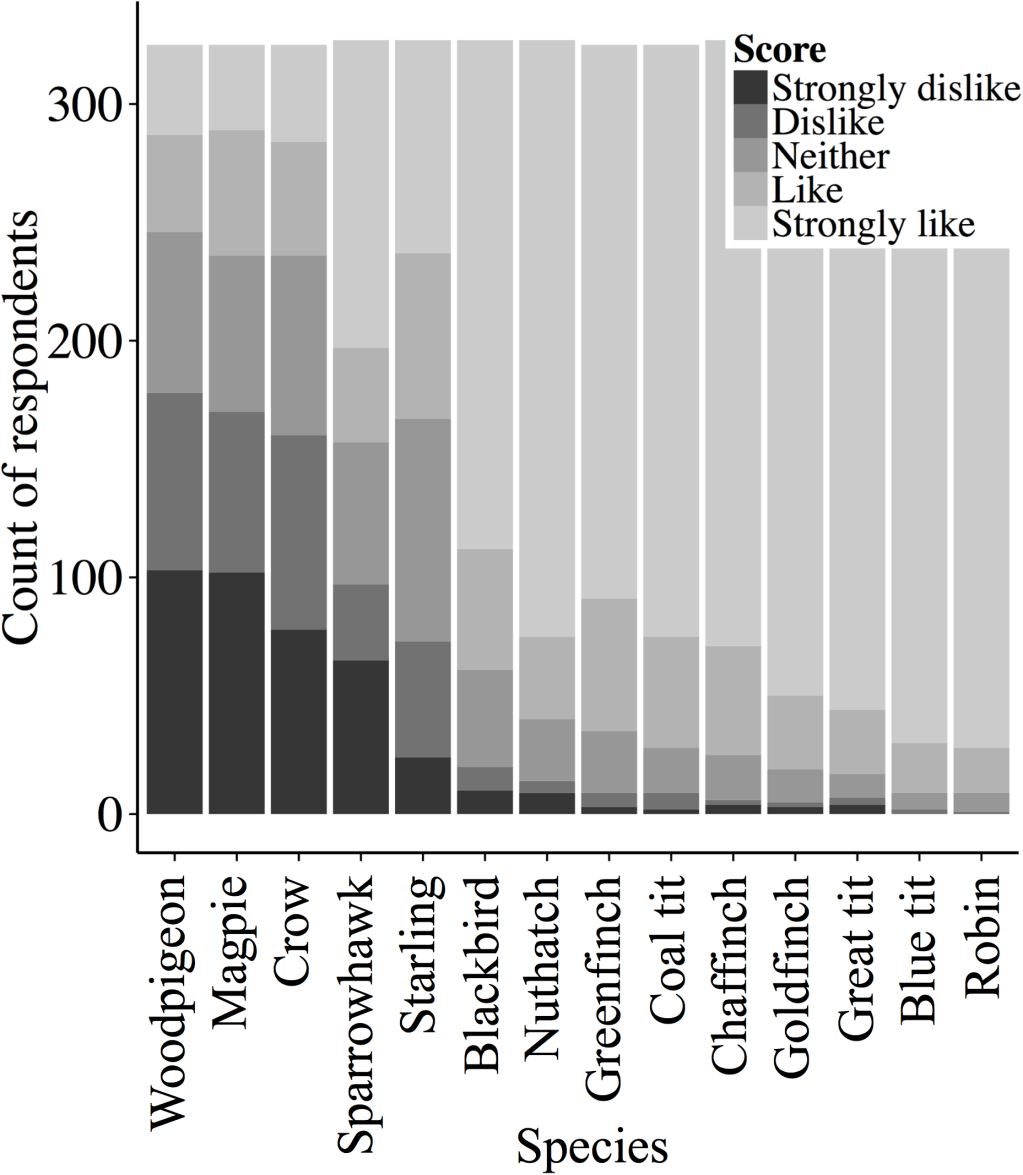
The likeability of 14 common garden species. Respondents were asked on a scale of 1–5 to rate how appealing they found each of the species (n = 327 ± 1.0 respondents). Species are ordered by the mean likeability score.

**Fig 2:**
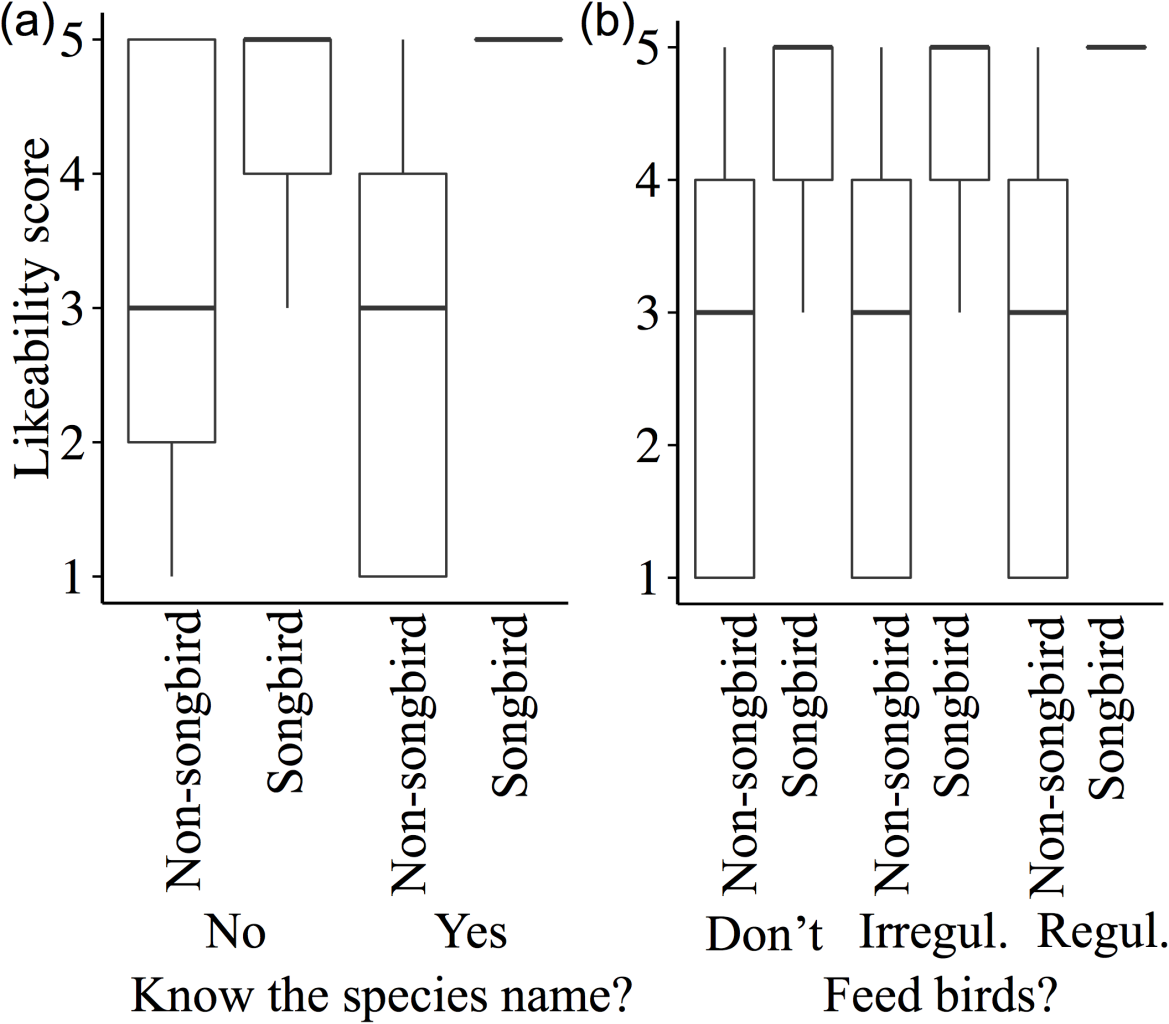
The interactions between (a) a person’s knowledge of the species name (Yes or No) or (b) how regularly they fed birds (Don’t; Irregularly “Irregul.”; Regularly “Regul.”), and whether the bird was a songbird or not (Songbird; Non-songbird), in determining the likeability of 14 common garden species (1 = strongly dislike, 5 = strongly like).

**Fig 3:**
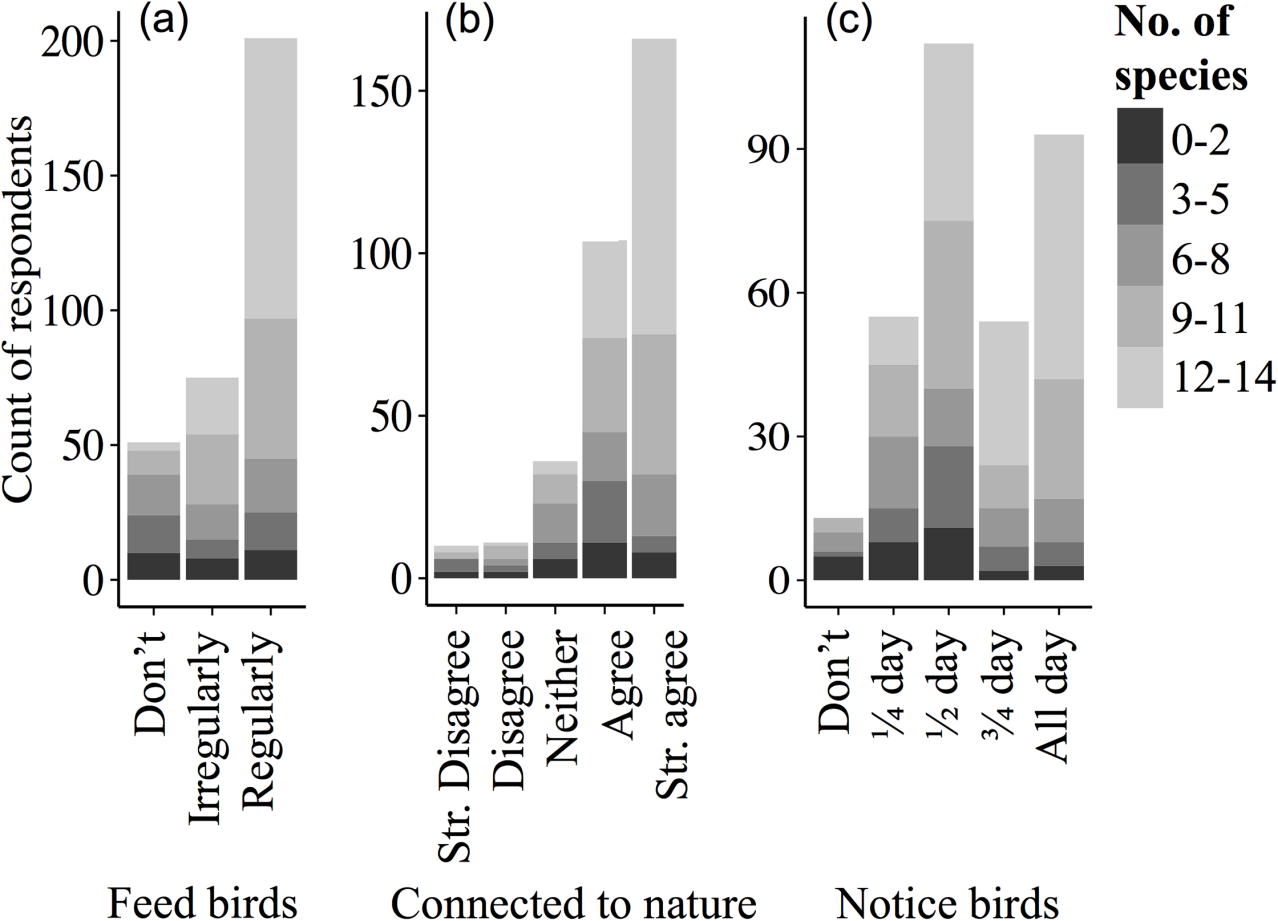
The relationship between the number of species that a person could name and (a) how regularly they fed birds, (b) how they agreed with the statement ‘I feel connected to nature when I watch birds in my garden’ (Strongly disagree “Str. disagree” to strongly agree “Str. Agree”), and (c) what proportion of the day they notice birds. The number of species that a respondent could name is also shown: 0–2, black; 3–5, dark grey; 6–8, medium grey; 9–11, medium light grey; 12–14, light grey.

Likeability varied markedly across the 14 species of common garden birds (Fig 1). The likeability of songbirds increased with knowledge of the species name and if people fed birds regularly, but decreased for non-songbirds (Fig 2). AICc showed support for a top model that contained the interactions between knowledge of the species name (ID) and how regularly people fed birds, with whether the bird was a songbird or not (taxa; Fig 2), the total period of the day that people noticed birds (notice), and their age (_W1_ = 0.44; Table 1). We found that the number of species a person could correctly identify was positively correlated with all three explanatory variables, how connected a person feels to nature when they watch birds in their garden, how regularly they put out food for birds, and for what proportion of the day they noticed birds (Table 2; Fig 3).

**Table 1:** Top candidate models and model averaged parameter estimates from the top models where ΔAICc <6, from a mixed model ordinal regression testing for the effect of every combination of explanatory factors and two interactions against how likeable people found 14 common garden bird species.

Model			*k*	AICc	ΔAICc	_Wi_
id*taxa*** + feed*taxa*** + id + feed + taxa + notice*** + connect***			7	8823.1	0.0	0.44
id*taxa + feed*taxa + notice + id + feed + taxa + connect + age			6	8824.0	0.9	0.29
id*taxa + feed*taxa + notice + id + feed + taxa + connect + sex			8	8825.1	2.0	0.17
id*taxa + feed*taxa + notice + id + feed+ taxa + connect + sex + age			9	8826.0	2.9	0.11
**Factor**	**Levels**	**Estimate (Std. Er.)**	**z value**		**RIV**	
Likeability	1|2	-1.1 (±0.5)	2.4*		1.00	
(dependent variable)	2|3	-0.2 (±0.5)	0.4			
	3|4	0.9 (±0.5)	1.9			
	4|5	1.6 (±0.5)	3.9			
Bird feeding (feed)	Irregularly	0.46 (±0.2)	2.5*		1.00	
	Regularly	0.54 (±0.2)	3.2**			
Species name (id)	Yes	0.4 (±0.1)	3.0**		1.00	
Taxa	Songbird	1.8 (±0.5)	3.3**		1.00	
Bird awareness	¼ day	0.01 (±0.2)	0.1		1.00	
(notice)	½ day	0.43 (±0.2)	2.6**			
	¾ day	0.45 (±0.2)	2.6**			
	All day	1.00 (±0.2)	5.7***			
Connected to nature	Disagree	0.9 (±0.1)	6.9		1.00	
(connect)	Neither	-0.2 (±0.1)	2.2			
	Agree	-0.1 (±0.1)	0.2			
	Strongly agree	-0.1 (±0.1)	0.6			
Age	40–60 years	-0.1 (±0.07)	1.5		0.39	
	>60 years	-0.02 (±0.07)	0.3			
Sex	Male	-0.01 (±0.02)	0.2		0.27	
Taxa * Species name	Songbird:Yes	1.0 (±0.1)	6.5		1.00	
Taxa * bird feeding	Songbird:Irregular	0.5 (±0.2)	2.2*		1.00	
	Songbird:Regular	0.9 (±0.2)	4.6***			

Models are ranked from lowest to highest ΔAICc (highest to lowest AICc weight, _Wi_).

Explanatory variables that contribute significantly (model-averaged coefficients) to the top-ranked model are marked as: *P < 0.05;

**P < 0.01;

***P < 0.001.

k is the number of model parameters.

RIV is the relative variable importance, which is the summed weigh of all models with a ΔAICc <6 that contain the variable of interest.

**Table 2:** ANOVA of the number of species that a person could identify against how regularly people notice and feed birds and how connected to nature they feel when they see birds in their garden. Age and gender were controlled for.

Factor	df	Sum sq.	F value	P value
Connection to nature	4	411.7	7.9	**<0.0001**
Feeding regularity	2	896.5	34.5	**<0.0001**
Notice birds	4	162.3	3.1	**0.02**
Gender	1	0.9	0.03	0.9
Age	2	0.4	0.07	0.8

### Nature dose quality or quantity

**Fig 4:**
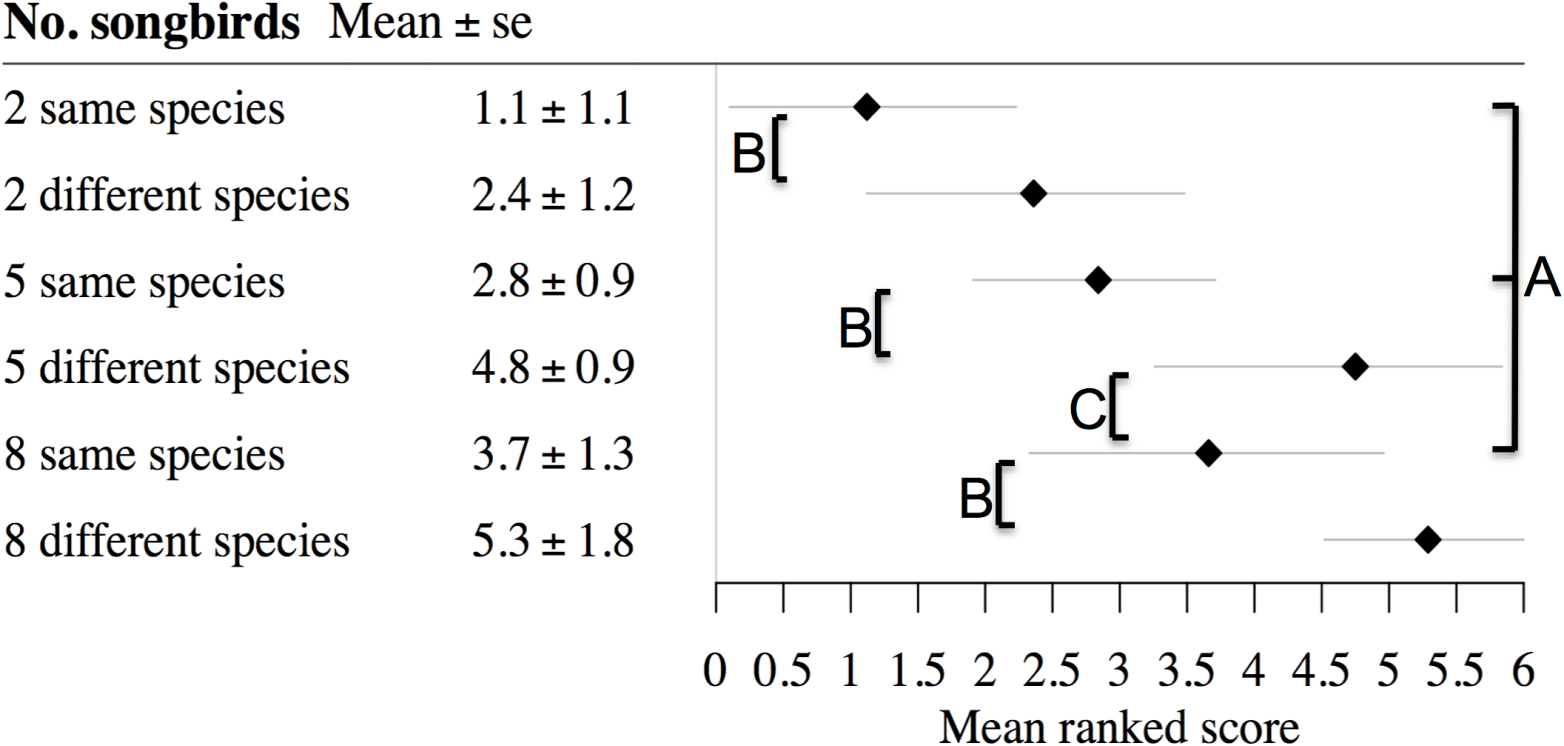
The relationship between species richness and individual abundance of birds that people preferred to see at their feeders. Respondents were shown six pictures of varying numbers of individuals and species of songbird, and asked to rank them in order from 1–6 with six being the picture that they were most satisfied with and one that they were least satisfied with. We show the mean ranked score and the standard error of each picture and key differences between pictures illustrating people preferring: A) more individuals of the same species than fewer, B) increased species richness across the same number of individuals, C) fewer individuals of more species, than more individuals of fewer species.

When shown different numbers of individuals of the same species, people consistently preferred to see more rather than fewer individuals (92% of respondents preferred 8 over 5 individuals, while 83% of respondents preferred 5 over 2 individuals; Table D in S1 File; Fig 4). A comparison of the same number of birds at the feeder (i.e. 8, 5 or 2) showed that people preferred to see individuals of different species over the same number of individuals of the same species (87%, 95%, 90% of respondents preferred to see 8, 5, or 2 species over an equivalent number of individuals of the same species; Table D in S1 File; Fig 4). People preferred to see five individuals all of different species over eight individuals of the same species (83% of respondents; Table D in S1 File; Fig 4). However, five individuals of the same species were preferred over two individuals of different species (74% of respondents; Table D in S1 File; Fig 4).

## Discussion

Garden bird feeding is a global phenomenon and one that continues to grow in popularity (reviewed by [18]). Providing food for garden birds clearly provides many people with a great deal of pleasure [16,17], while the provision of large quantities of supplementary food across many urban and rural landscapes has implications for both avian demographics and welfare (e.g. [24,25,40]). As a consequence, understanding what people want to see in their garden has implications for economics, conservation and ultimately, how we manage green spaces to connect people to nature and so maximise the associated benefits.

We show that there is substantial variation in likeability across 14 common UK garden species, with songbirds being preferred over non-songbirds. Songbirds tend to be unobtrusive, brightly coloured and are rarely a source of human-avian conflict. They also engage in behaviours that people tend to find interesting, and watching them is recognised as contributing towards recovery from attention deficit fatigue [1,41]. The importance that people attach to garden songbirds is perhaps illustrated in a recent poll selecting the robin *Erithacus rubecula* as the UK’s favourite bird [42]. This concurred with the results in this study (Fig 1). General support for songbirds is perhaps best portrayed through the wide variety of commercial bird food available that targets these species. Conversely, the four non-songbird species were thought of less positively. Within urban areas where relatively dense human and avian populations coexist, activities by a small number of species such as calling and singing, aggression and causing damage to properties, can result in conflict with their human neighbours [32,43]. Interactions with species that are thought of less positively are unlikely to result in the provision of the same well-being benefits, and may even cause human-avian conflict. Starlings were thought of least positively of the songbirds, possibly resulting from their tendency to strip feeders of food and out compete other songbirds.

We found that people’s positive or negative feelings about a species increased with some knowledge of that species, assessed here by whether they could name it or not. This suggests that a person’s experience of a species is a strong contributor towards the well-being they receive from observing it. This has ecological and economic implications because the general attitude of residents to a species will affect how people interact with it, such as the provision of, or removal of, food and nesting spaces, and scaled-up can have consequences for both bird population dynamics and human well-being. Thus, species that visit feeders are more likely to provide more well-being benefits than those that ‘skulk’ in the bushes, because the former are the species that people see and are familiar with. However, species likeability should not be confused with the value a person places on the species in itself and the ecological role it plays in the environment. Knowledge concerning the avifauna in an urban green space can also enhance the cultural value that people place on that area [44]. Worryingly, studies indicate that many people living in urban areas are unaware of the nature that is around them [6,45]. This study suggests that those people who are less aware and interested in interacting with the birds around them may receive reduced well-being benefits from seeing the same birds compared to a person who is more orientated towards interacting with them.

We found a correlation between the number of species that a respondent could correctly identify and how connected to nature they felt when they watched garden birds (Fig 3). Although this does not demonstrate a casual relationship it does suggest that the two may be linked. Being connected to nature is an important step in mediating the extinction of experience (the progressive loss of human interactions with nature), in raising people’s awareness of the nature around them, and in understanding conservation issues in the wider world [46,47]. Gardens can evoke strong feelings of ownership and sense of place [48], with many urban dwellers spending significant amounts of time there [49,50]. Therefore the nature that is found in gardens may act as an important stepping-stone in providing this connection, because it is the nature that people will experience on a daily basis and so be more familiar with. As such, relevant stakeholders and initiatives should focus on increasing urban residents’ awareness of the nature around them. Citizen science initiatives have shown that this can be an effective approach to increasing people’s nature awareness [51].

We showed respondent’s pictures of varying numbers of individuals and species of songbird at garden bird feeders. Assuming that people gain increased well-being benefits from being ‘more satisfied’ with the birds they see at their feeders we tested how benefits vary from seeing different nature intensities (i.e. quantity and quality, respectively). A key question in environmental psychology and urban planning is understanding the role that species richness plays in people’s relationship with urban nature (e.g. [45]). Research suggests that people prefer greater species richness, but are generally unable to recognise it, with perceptions of biodiversity being only weakly linked to actual biodiversity [6,32,45]. Exploring these relationships with birds at supplementary feeders largely overcomes this issue, because by their nature feeders tend to be placed in locations where people can most easily see birds. It is also possible to distinguish between many species, allowing people with limited knowledge to appreciate species richness, thus the gap between the actual and perceived richness at feeders is likely to be much reduced. We found that consistently people preferred to see more individuals of the same species than fewer (i.e. nature intensity quantity), and increased species richness across the same number of individuals (i.e. nature intensity quality). This study joins the growing body of evidence that suggests that people receive increased well-being benefits from seeing more birds and that increased species richness increases the benefits [4,6,32].

Previous studies have found a positive relationship between self-reported well-being and either actual or perceived species richness [4,6,32]. Here for the first time we provide evidence that the relationship between nature intensity quality and quantity changes as the number of individual birds increase (Fig 4). Overwhelmingly respondents preferred to see species richness to an increased abundance of individuals, which suggests that the well-being benefits provided per bird are dependent not solely on that bird but are magnified depending on the richness of the avian assemblage in that area. However, there is likely a threshold to the benefits that people receive as individual abundance and species diversity continue to increase, after which further increases in biodiversity may even cause reduced well-being through human-avian conflict [32]. Understanding the shape of the dose-response relationship between biodiversity and psychological well-being benefits is key for developing best management practices in order to maximise these benefits for personal and social well-being.

## Conclusion

This study provides evidence that the well-being benefits that people receive from interacting with the birds in their garden is dependent on their familiarity with different species, and that these benefits are enhanced by increased species richness. Attention should be given to strategies that focus on increasing the diversity of songbirds within urban green spaces, along with increasing the ability of recreational green space users to recognise different components of the natural environment. Combined these approaches will enhance both the well-being benefits that people receive from interacting with nature and the biological complexity of urban green spaces. Bird feeders provide a powerful tool for people to engage with the natural world in their own garden, and so act as an important stepping-stone for a wider connectedness to nature. People with a greater connection to nature are more likely to be aware of, and support, conservation issues in the wider landscape (reviewed in [52]). So feeding birds can be seen as an important tool in reconnecting people to the natural world, so helping to mediate the extinction of experience.

## Supporting Information

S1 File Test of whether two methods of data collection were comparable (Appendix A). Likeability and knowledge of 14 common garden species **(Table A)**. Summary of questions asked in survey **(Table B).** Demographic breakdown of the respondents, with comparative nationwide data from UK Census 2011 **(Table C).** Summary of the count of respondents who rank ordered their preference of pictures of different numbers of individuals and species at a bird feeder **(Table D**). Exploring the relationship between nature dose quality and quantity **(Figure A).**
(DOCX)
