# Supplementary material for: Likeability of Garden Birds: Importance of Species Knowledge & Richness in Connecting People to Nature
Source: PLoS One. 2015 Nov 11;10(11):e0141505. doi: 10.1371/journal.pone.0141505 (PMC4641628; doi:10.1371/journal.pone.0141505)
Supplement: S1 File — Likeability and knowledge of 14 common garden species (Table A). Summary of questions asked in survey (Table B). Demographic breakdown of the respondents, with comparative nationwide data from UK Census 2011 (Table C). Summary of the count of respondents who rank ordered their preference of pictures of different numbers of individuals and species at a bird feeder (Table D). Exploring the relationship between nature dose quality and quantity (Figure A). (DOCX) [file pone.0141505.s001.docx]

**Supplementary Information**

**Appendix A.** **We tested whether our two methods of data collection were comparable.** We did this by pooling the answers from all nine Likert statements, before using the ‘ordinal’ package to build an ordinal mixed effect model to test whether there was a difference in responses to statements between our two methods of data collection (two-level factor). Each statement was assigned an identification number and this was included as a random factor. We did not find any difference in responses across our two methods of data collection (coefficient = 0.02 (±0.04 SD), *P* = 0.7). Both methods of data collection had a high response rate (90 and 94% respectively) and as a consequence we did not test for non-response bias.

The following includes relevant sections that were used in the analysis reported here.

**Table A. Likeability and knowledge of 14 common garden species**. Respondents were asked to rate on a scale of 1-5 from strongly dislike to strongly like, how appealing they found pictures of 14 common garden species. Pictures were shown in the following order, and covered seven rows by two columns over two A4 pages. All photos were approximately 45 mm high and 60 mm wide. In each photo a full body shot showed the bird perched, fully occupying the centre of the picture with all distinguishing characteristics being clearly visible. Under each picture the respondent was first asked to score on a scale of 1-5 from strongly dislike to strongly like, how appealing they found each picture. Under the score they were asked to add the species name if they knew it (knowledge). Here we also show the mean score for each species.

| **Common name** | **Latin name** | **Mean** | **Likeability** | | | | | **Knowledge** | |
| --- | --- | --- | --- | --- | --- | --- | --- | --- | --- |
|  |  | **(± SD)** | **1** | **2** | **3** | **4** | **5** | **No** | **Yes** |
| Robin | *Erithacus rubecula* | 4.9 (±0.4) | 0 | 1 | 8 | 19 | 305 | 25 | 308 |
| Blackbird | *Turdus merula* | 4.4 (±1.0) | 11 | 11 | 41 | 51 | 220 | 39 | 295 |
| Blue tit | *Cyanistes caerule* | 4.9 (±0.4) | 0 | 2 | 7 | 23 | 301 | 50 | 283 |
| Great tit | *Parus major* | 4.8 (±0.6) | 4 | 3 | 10 | 28 | 288 | 126 | 207 |
| Chaffinch | *Fringilla coelebs* | 4.7 (±0.7) | 4 | 2 | 21 | 46 | 260 | 107 | 226 |
| Sparrowhawk | *Accipiter nisus* | 3.4 (±1.5) | 66 | 33 | 63 | 40 | 131 | 182 | 151 |
| Nuthatch | *Sitta europaea* | 4.6 (±0.9) | 9 | 6 | 27 | 35 | 256 | 212 | 121 |
| Starling | *Sturnus vulgaris* | 3.5 (±1.2) | 24 | 50 | 95 | 72 | 92 | 92 | 241 |
| Coal tit | *Periparus ater* | 4.6 (±0.7) | 2 | 7 | 21 | 47 | 254 | 130 | 201 |
| Magpie | *Pica pica* | 2.6 (±1.4) | 102 | 69 | 68 | 56 | 36 | 62 | 269 |
| Greenfinch | *Carduelis chloris* | 4.6 (±0.8) | 3 | 7 | 26 | 58 | 237 | 157 | 174 |
| Crow | *Corvus corone* | 2.7 (±1.3) | 74 | 84 | 76 | 52 | 41 | 113 | 218 |
| Goldfinch | *Carduelis carduelis* | 4.8 (±0.7) | 3 | 3 | 14 | 32 | 279 | 158 | 173 |
| Woodpigeon | *Columba palumbus* | 2.5 (±1.3) | 103 | 77 | 71 | 42 | 39 | 48 | 284 |

**Table B. Summary of questions asked in survey.**  We established baseline information about household bird related activities and demographics.

| **Question** | **Response options** |
| --- | --- |
| 1. How often do you *usually* put out food for birds? (Please tick one) | Daily, Weekly, Monthly, Less than once a month, Never |
| 1. In which season(s) do you *usually* put out food for birds? (Please tick as appropriate) | Winter, Spring, Summer, Autumn |
| 1. When do you notice birds where you live and/or work? (please tick as appropriate) | Morning, Lunchtime, Afternoon, Evening, I don’t really notice birds |
| 1. I feel connected to nature when I watch birds in my garden | Five-point scale from strongly dislike to strongly like |
| 1. What is your age range? | Increments of five years from 20 until 70+ years |
| 1. What is your gender? | Female, Male |
| 1. What is your postcode? |  |
|  |  |

**Table C. Demographic breakdown of the respondents, with comparative nationwide data from UK Census 2011.** We also show the percentage of respondents that answered each factor level. The total number of survey respondents *n* = 336.

| **Gender (%)** | | **Age (years; %)** | | **Feeding (%)** | **Notice (%)** | **Connect (%)** |
| --- | --- | --- | --- | --- | --- | --- |
| *Survey* | *National* | *Survey* | *National* | Don’t: 12.0 | 0: 4.2 | 1: 3.3 |
| Male: 40.0 | 49.1 | 20 to 40: 14.6 | 35.2 | Irregularly: 24.4 | 1: 16.7 | 2: 10.7 |
| Female: 60.0 | 50.9 | 40 to 60: 38.8 | 35.3 | Regularly: 60.7 | 2: 32.7 | 3: 10.7 |
|  |  | >60: 45.6 | 29.5 |  | 3: 16.1 | 4: 30.2 |
|  |  |  |  |  | 4: 27.4 | 5: 47.9 |

**Table D.** Summary of the count of respondents who rank ordered their preference of pictures of different numbers of individuals and species at a bird feeder. Pictures were ranked from 1-6 with one being the picture that they were least satisfied with and six being the picture that they were most satisfied with.

| **No. of songbirds at feeder** | **Rank order** | | | | | |
| --- | --- | --- | --- | --- | --- | --- |
|  | **1** | **2** | **3** | **4** | **5** | **6** |
| 2 of same species | 161 | 21 | 15 | 5 | 7 | 2 |
| 2 of different species | 27 | 154 | 20 | 34 | 7 | 9 |
| 5 of same species | 9 | 35 | 169 | 32 | 5 | 1 |
| 5 of different species | 4 | 6 | 8 | 30 | 182 | 29 |
| 8 of same species | 9 | 18 | 32 | 144 | 42 | 6 |
| 8 of different species | 12 | 3 | 3 | 4 | 17 | 211 |


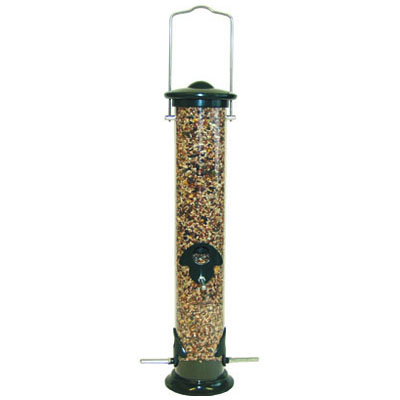


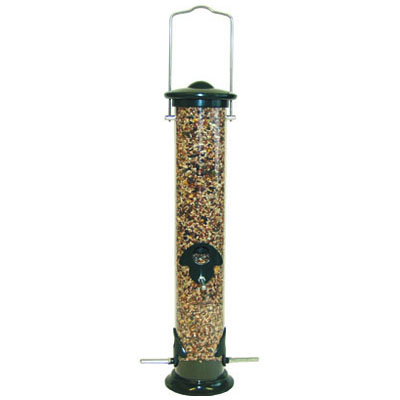

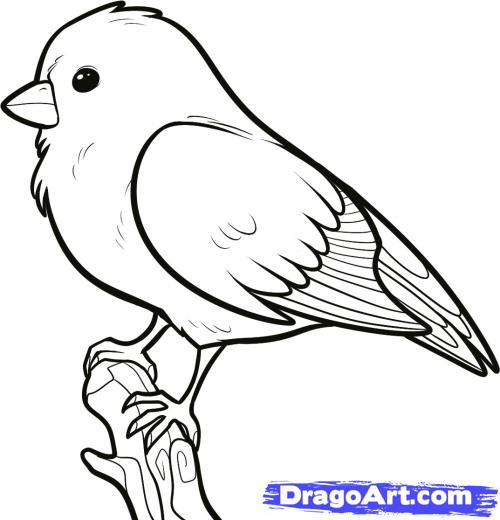
a) b)


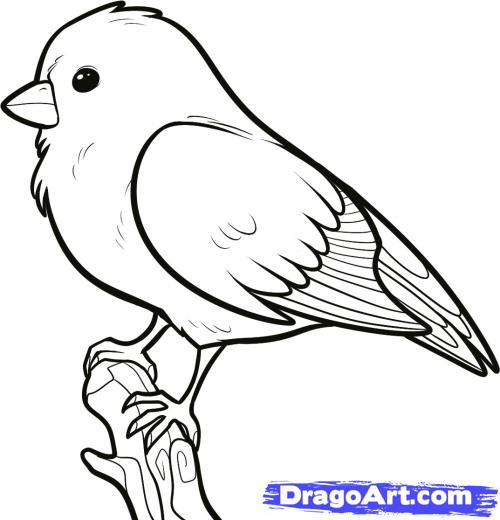


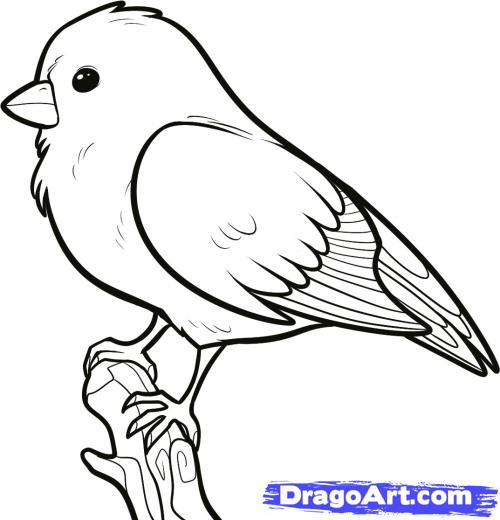

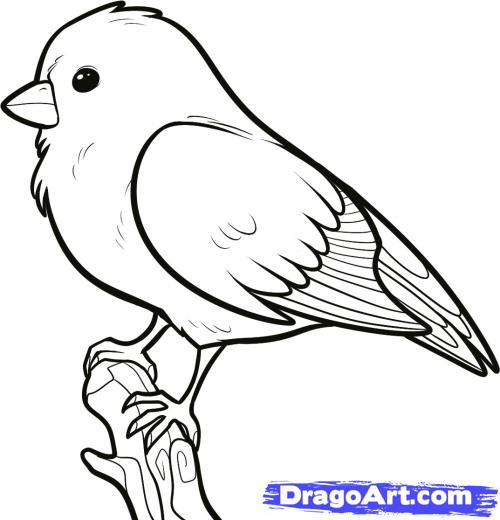


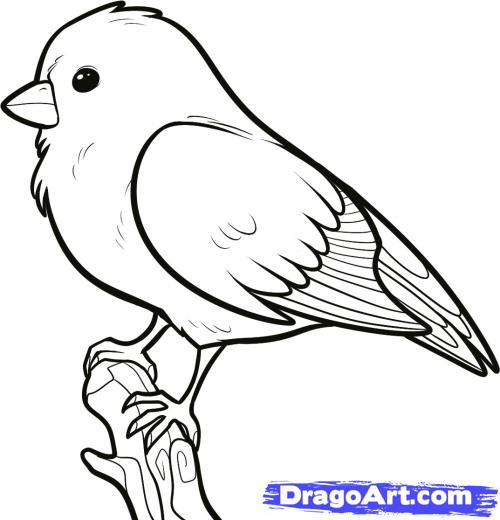

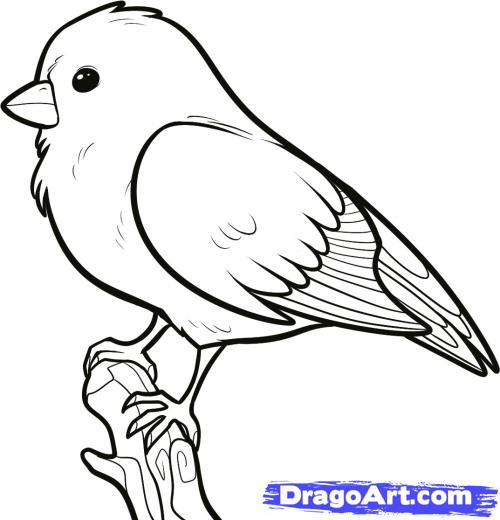


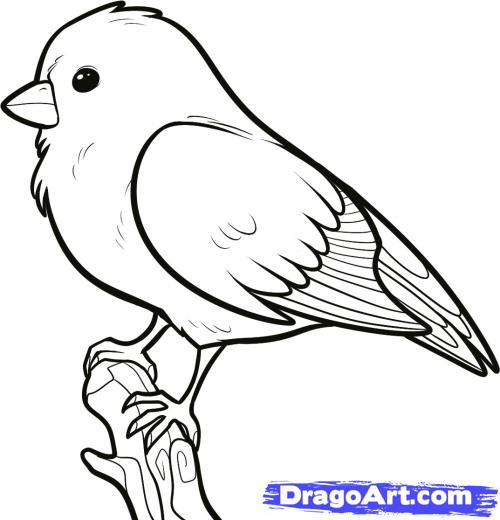

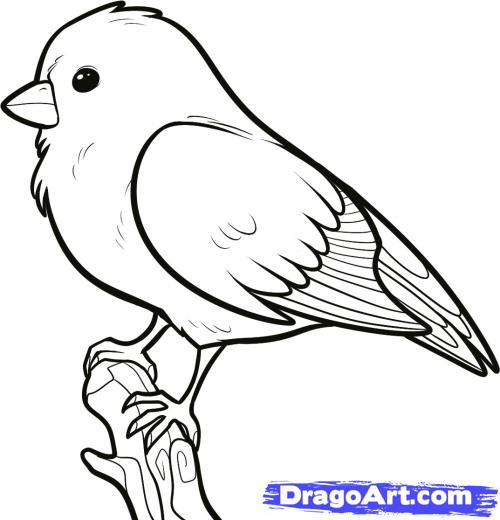


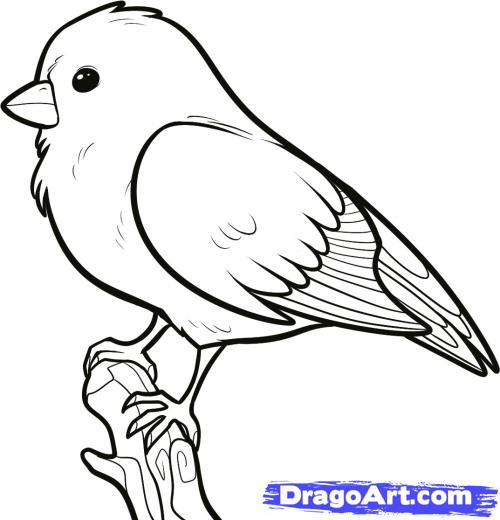

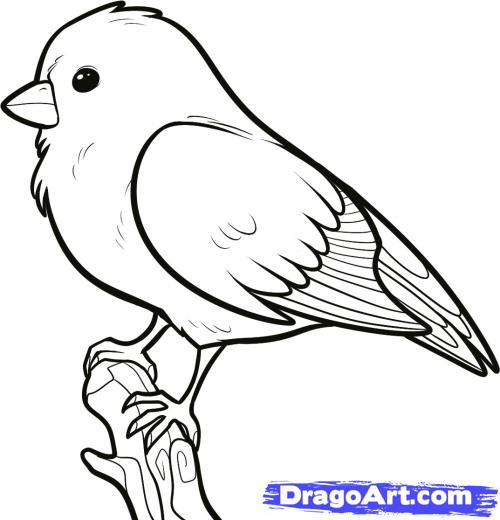


Score: Score:


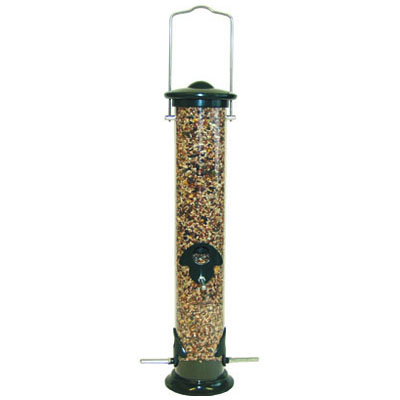

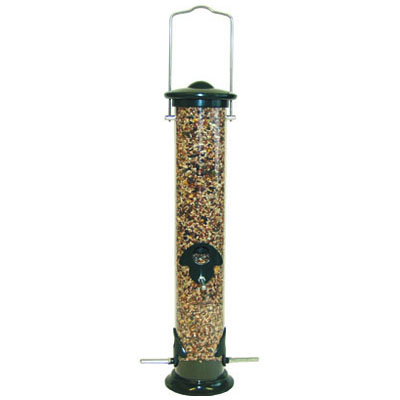
c) d)


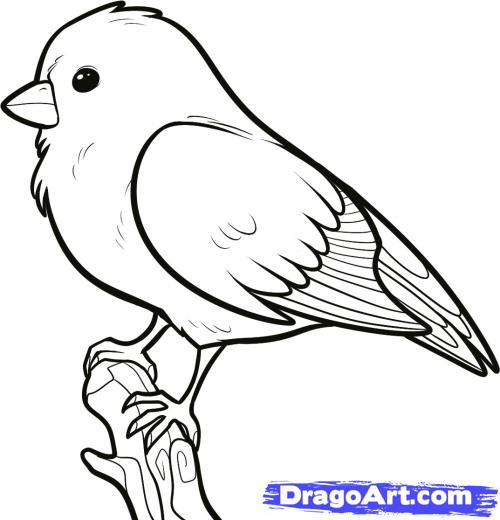


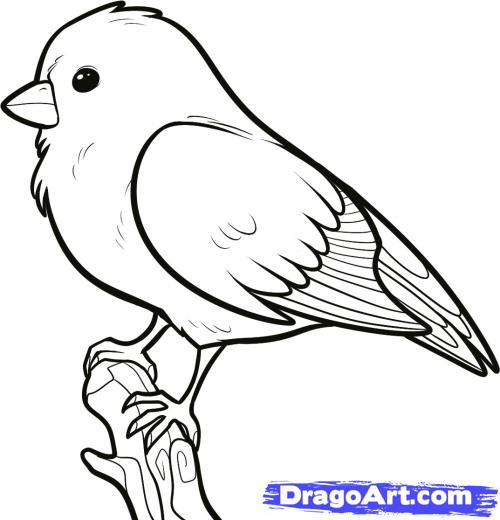

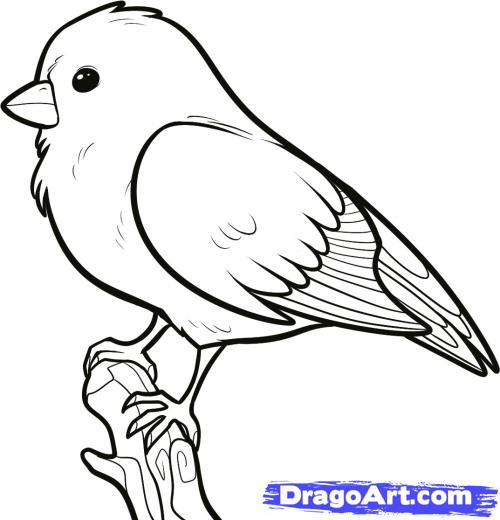


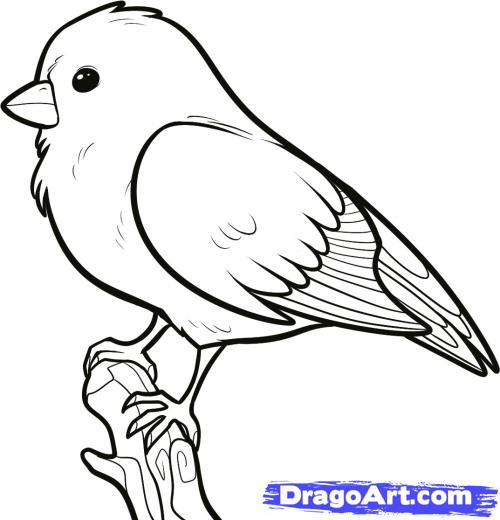


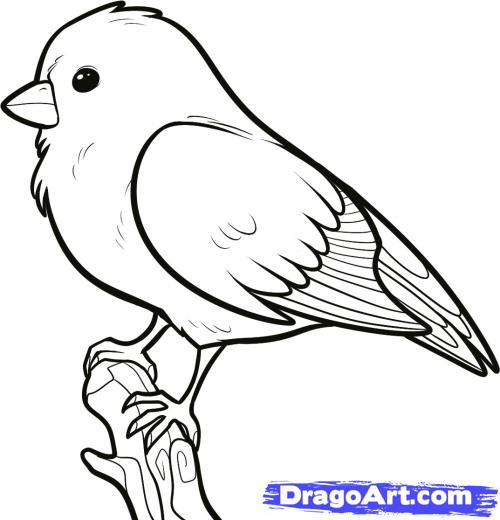


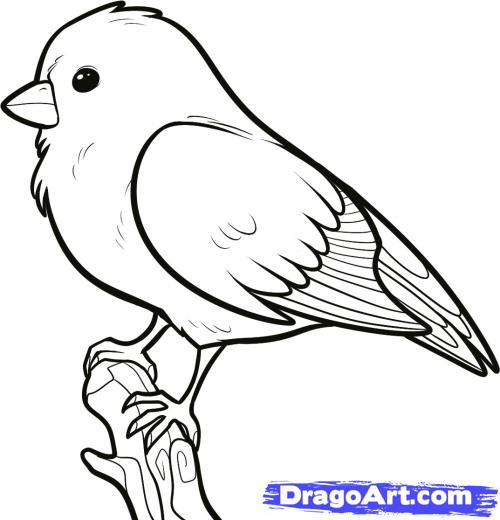

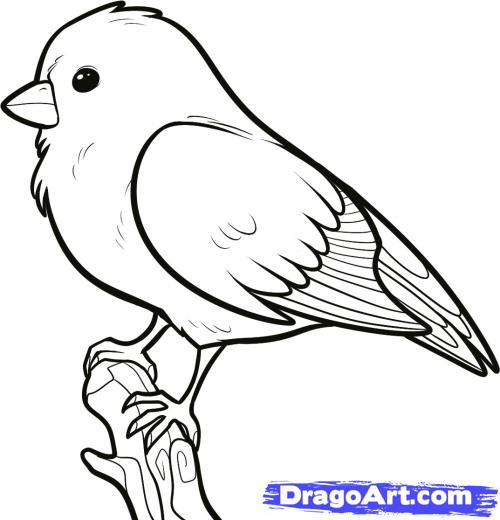


Score: Score:


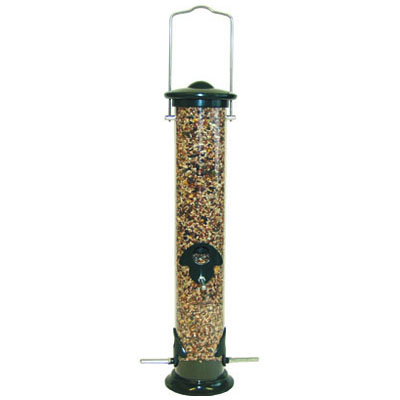

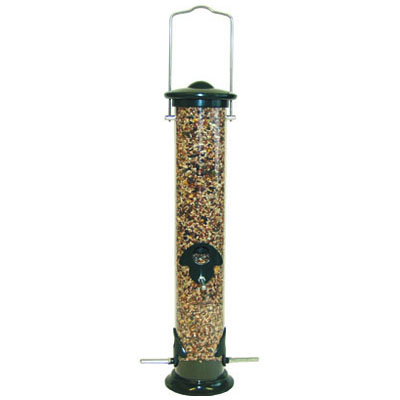


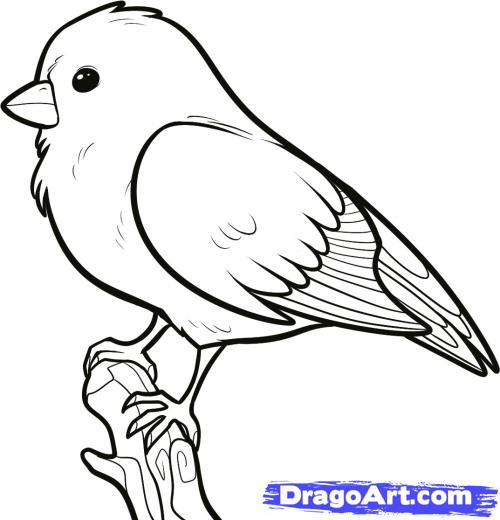

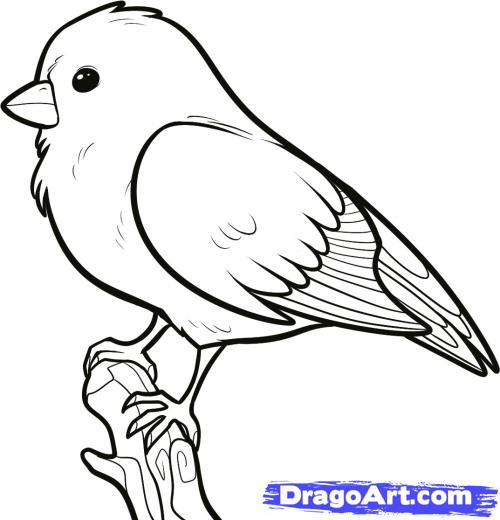
e) f)


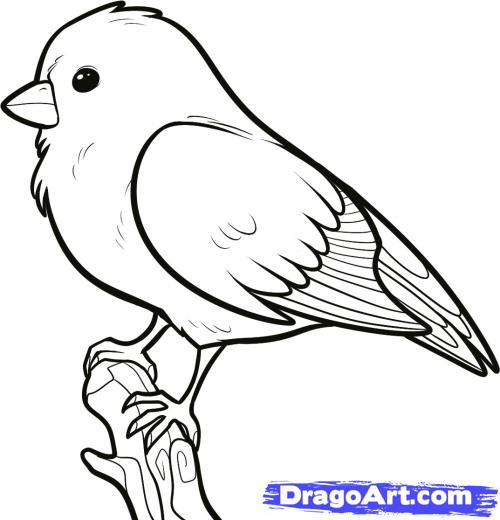

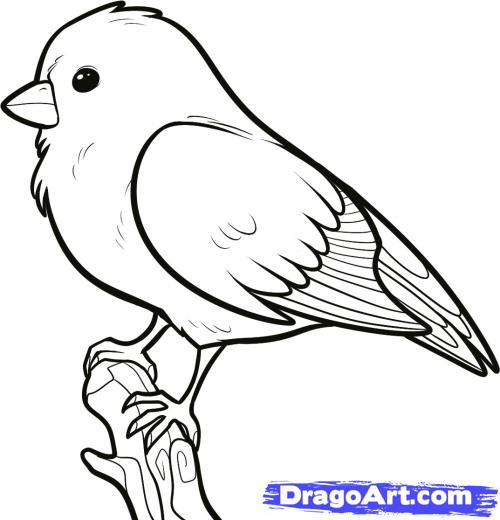


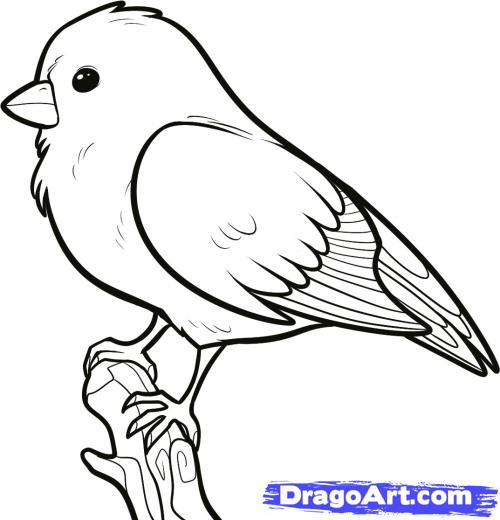

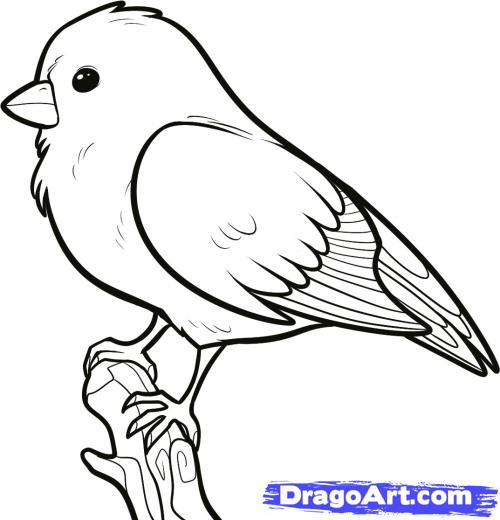


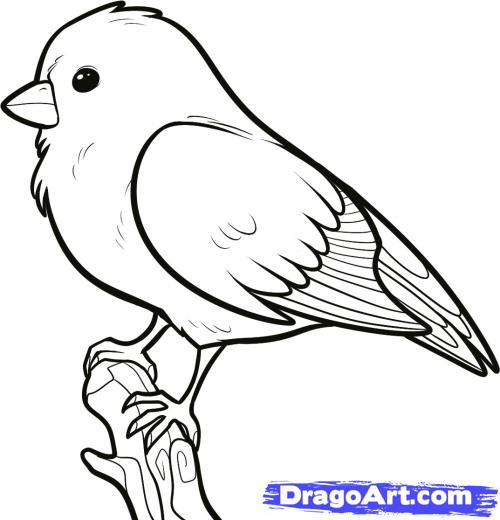

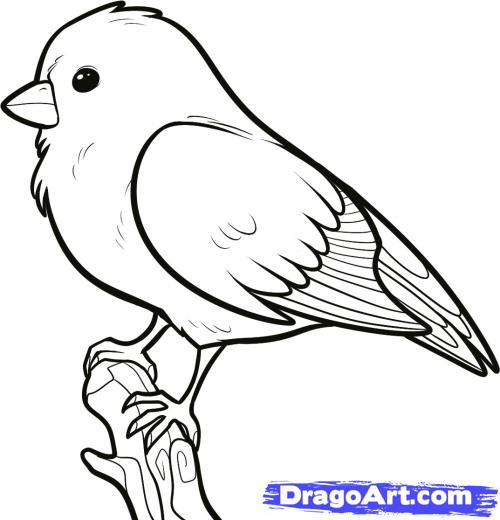

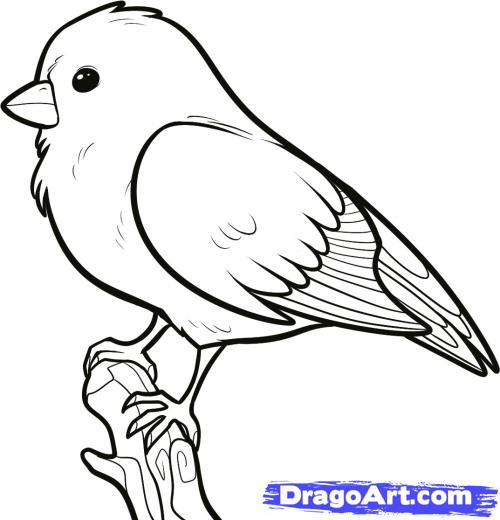


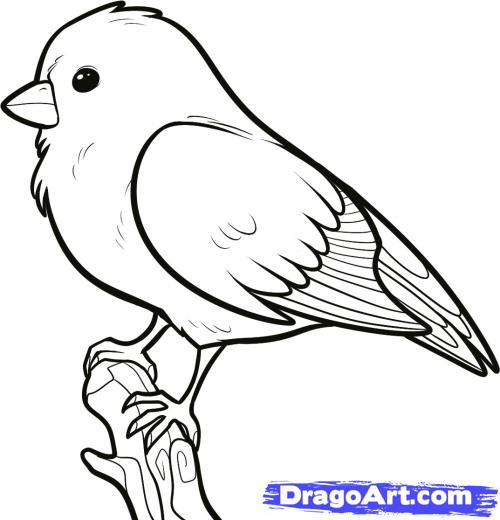


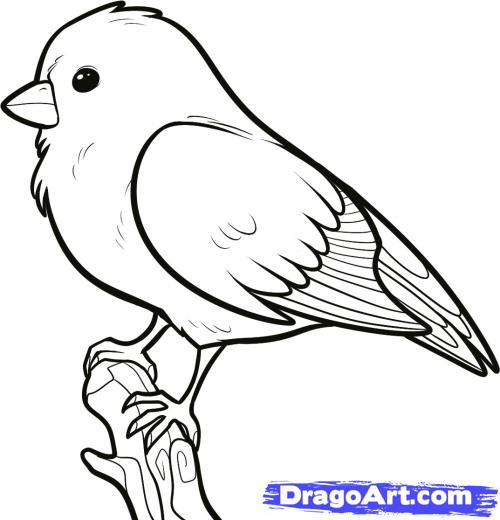

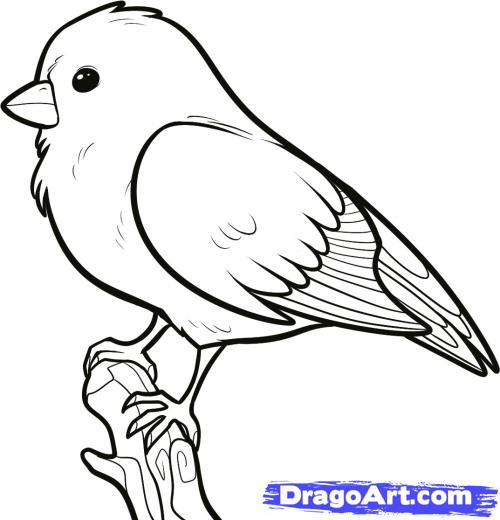

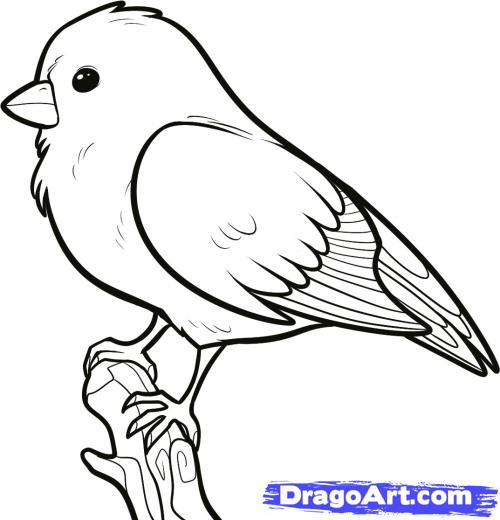


Score: Score:

**Figure A. Exploring the relationship between nature dose quality and quantity.** Respondents were told that each coloured bird represents a different species. They were asked to think about the birds that visit their feeders, and then to rank the following pictures in order from 1-6 with 6 being the picture that they were most satisfied with and 1 being the picture that they were least satisfied with. The pictures were shown as above.
